# Continuous Flow Photochemical and Thermal Multi-Step Synthesis of Bioactive 3-Arylmethylene-2,3-Dihydro-1*H*-Isoindolin-1-Ones

**DOI:** 10.3390/molecules24244527

**Published:** 2019-12-11

**Authors:** Saira Mumtaz, Mark J. Robertson, Michael Oelgemöller

**Affiliations:** 1 College of Science and Engineering, James Cook University, Townsville, QLD 4811, Australia; 2 Department of Organic and Macromolecular Chemistry, Ghent University, Krijgslaan 281 S4-bis, 9000 Gent, Belgium

**Keywords:** photochemistry, photodecarboxylation, phthalimide, continuous-flow photochemistry, multi-step synthesis, arylmethylene isoindolinones

## Abstract

An effective multi-step continuous flow approach towards *N*-diaminoalkylated 3-arylmethylene-2,3-dihydro-1*H*-isoindolin-1-ones, including the local anesthetic compound AL-12, has been realized. Compared to the traditional decoupled batch processes, the combined photochemical–thermal–thermal flow setup rapidly provides the desired target compounds in superior yields and significantly shorter reaction times.

## 1. Introduction

**Scheme 1:**
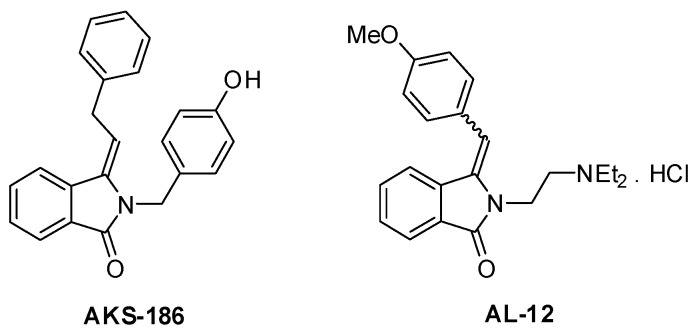
Examples of bioactive 3-arylmethylene-2,3-dihydro-1*H*-isoindolin-1-ones.

Due to their broad biological activities, the synthesis of 3-arylmethylene-2,3-dihydro-1*H*-isoindolin-1-ones has been widely studied [1,2]. Among the many synthetic pathways developed, the photodecarboxylative benzylation of phthalimides represents a mild and efficient access to these important target compounds [3,4]. Subsequently, this photobenzylation has been successfully applied as a key step in the synthesis of the cardiovascularly active AKS-186 and the local anesthetic AL-12 (Scheme 1) [5,6]. The syntheses protocols involved utilize conventional step-by-step batch procedures. Examples of photodecarboxylations involving phthalimides in circulating or continuous-flow reactors have also been realized [7,8].

Flow chemistry allows for the continuous manufacturing of target compounds under controlled conditions [9,10]. This technology has proven particularly beneficial for photochemical transformation as it permits effective light utilization and protection of photoactive products [11,12,13]. Flow operation furthermore enables easy coupling of individual reaction steps without the need for isolation and purification of intermediates (‘telescoping’) [14]. A growing number of multi-step processes incorporating a photochemical key-step have already been described [15]. Following this development, we have successfully established a three-step one-flow process for the continuous synthesis of a series of *N*-diaminoalkylated 3-arylmethylene-2,3-dihydro-1*H*-isoindolin-1-ones.

## 2. Results

### 2.1. Synthesis Optimization

**Scheme 2:**
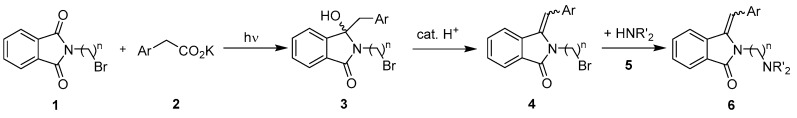
Synthesis of *N*-diaminoalkylated 3-arylmethylene-2,3-dihydro-1*H*-isoindolin-1-ones**.**

The three-step synthesis of the desired *N*-diaminoalkylated 3-arylmethylene-2,3-dihydro-1*H*-isoindolin-1-ones **6** is outlined in Scheme 2. Initial photobenzylation of the readily available *N*-(bromoalkyl)phthalimides (**1**) with arylactetates (**2**) produces the corresponding benzylated hydroxyl phthalimidines (**4**) as key intermediates. Subsequent acid-catalyzed dehydration [16], followed by amination with the corresponding secondary amines (**5**) [17] yields the desired target compounds **6**.

Under batch conditions, the photodecarboxylation, dehydration, and amination steps were performed in three different solvents, i.e., acetone/pH 7 buffer, dichloromethane and DMF [6]. To avoid a switch of solvents under continuous flow conditions, a common solvent for all three reaction steps was desirable. *N*-(2-Bromoethyl)phthalimide (**1a**), phenylacetate (**2a**) and diethylamine (**5a**) were thus chosen as model reagents for a solvent study. Following the original reaction protocols, each step for the synthesis of **6a** was conducted separately in batch in acetone, DMF and/or acetonitrile, respectively (Table 1). Dichloromethane was omitted due to the poor solubility of most reagents in this solvent. For the photodecarboxylation, pH 7 buffer (33 vol%) was used as a co-solvent to minimize side-reactions (entries I–III) [3]. Overall, acetonitrile was found to be the most suitable solvent as it gave acceptable to good conversions for all three reaction steps without the formation of noticeable by-products (entries II, V, and VIII). The acid catalyzed dehydration step produced large amounts of the aldol-condensation product of acetone [18], which was difficult to remove (entry IV).

### 2.2. Flow Reactor Design

**Figure 1:**
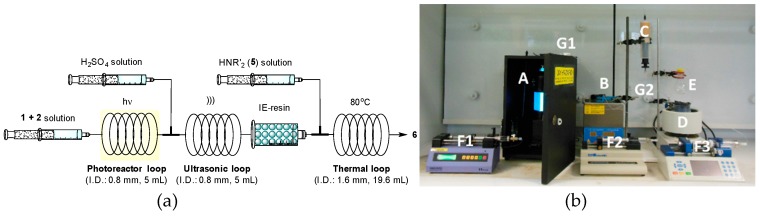
(**a**) Flow scheme; (**b**) Actual in-house multi-step flow reactor module (A: photo reactor, B: ultrasonic reactor, C: ion exchange resin cartridge, D: thermal reactor, E: product collection flask, F1–3: syringe pumps, G1/2: T-junctions).

Photodecarboxylative additions in continuous-flow mode were initially investigated using a previously described in-house capillary reactor [19]. The reactor used a transparent fluorinated ethylene propylene (FEP) capillary wrapped around a Pyrex body with a single 8 W UVB fluorescence tube at its center. This photoreactor module was subsequently coupled via a FEP T-junction with a thermal loop. Following a reactor concept reported by Horie and co-workers [20], the capillary coil of this thermal module was placed in an ultrasonic bath in order to prevent precipitation of the rather non-polar dehydration products **4**. Finally, the reactor system was expanded into a three component setup coupled through FEP T-junctions (Figure 1). Micro-FEP tubing was used for the first and the second loop (each with an I.D. of 0.8 mm, a length of 10 m, and an internal volume of 5 mL), whereas a wider tubing diameter was chosen for the third module (I.D. of 1.58 mm, length of 10 m, and internal volume of 19.6 mL). Reactant solutions were injected into the reaction streams with syringe pumps between each reactor. A strongly basic ion exchange resin cartridge (ca. 50 mL) was placed between the second and third reactor module to remove excess of acid after the dehydration step. A similar approach was described by DeLaney et al. for the removal of base [21]. Heating was achieved in the third step by submerging the capillary coil in a water bath. The reactor components are described in detail in the Supplementary Materials.

### 2.3. Continuous-Flow Operations

#### 2.3.1. Photodecarboxylation

**Scheme 3:**
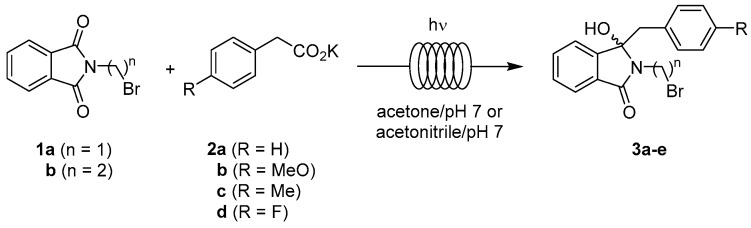
Photodecarboxylative additions of arylactetates to *N*-(bromoalkyl)phthalimides.

Representative examples of the photodecarboxylative addition reaction were initially carried out under flow conditions with the aim to obtain high to complete conversions (Scheme 3 and Table 2). Previously degassed mixtures of the corresponding *N*-(bromoalkyl)phthalimides (**1a** and **b**) and arylactetate (**2a**–**d**) were injected into the capillary photoreactor and the product solution was collected externally in a round bottom flask. An excess of arylacetate was utilized to suppress competing simple decarboxylations (-CO_2_H ↔ -H exchange) [22]. When pH 7 buffer-acetone was used as the reaction medium, complete conversions were achieved with a residence time of 20 min (corresponding to a reagent flow rate of 0.25 mL/min) and the benzylated hydroxyl phthalimidines **4a**–**e** were isolated in good to high yields of 80%–95% (entries I–V). Prolonged residence times of 30 min (reagent flow rates of 0.17 mL/min) were required in pH 7 buffer-acetonitrile solutions but likewise furnished the desired target compounds **4a**–**e** in high yields of 83%–91% (entries VI–X).

#### 2.3.2. Photodecarboxylation-Dehydration Coupling

**Scheme 4:**
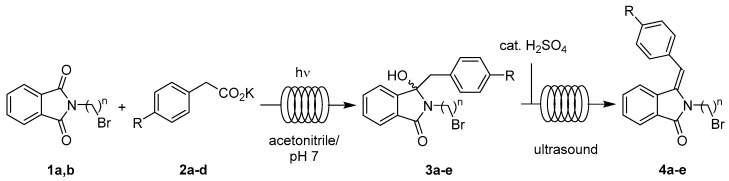
Photodecarboxylation with subsequent dehydration.

The photoaddition step was subsequently coupled with the first thermal reactor loop (Scheme 4 and Table 3). The initial photoreaction mixture in pH 7 buffer-acetonitrile was pumped through the photoreactor module at 0.17 mL/min, resulting in a residence time of 30 min. A 1:1 mixture of a 10 M solution of sulfuric acid and acetonitrile was injected in a FEP T-junction into the effluent at a flow rate of 0.17 mL/min. The combined reaction stream was pumped through a FEP capillary submerged in an ultrasonic bath and the product mixture was collected externally in a flask. After workup and isolation, the corresponding 3-arylmethylene-2,3-dihydro-1*H*-isoindolin-1-ones **4a**–**e** were obtained in good to high yields of 83%–94% with near complete *E*-selectivity (*E*:*Z* ≥ 9:1), as confirmed by ^1^H-NMR spectroscopic analysis [16].

#### 2.3.3. In Series Multistep Flow Operation

**Scheme 5:**
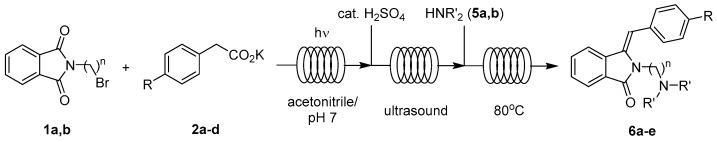
Photodecarboxylation with subsequent dehydration and amination.

The entire three-step procedure was consequently realized in the combined photochemical–thermal–thermal flow setup shown in Figure 1 (Scheme 5 and Table 4). As before, the initial photodecarboxylative addition was performed in the photoreactor and the subsequent dehydration in the thermal ultrasonic module. The effluent stream from the dehydration loop entered a cartridge containing strongly basic Dowex 1-X8 ion exchange resin against gravity from the bottom, effectively neutralizing excess amounts of acid. A solution of the corresponding amine **5a** or **b** in acetonitrile was subsequently introduced into the reagent stream in a FEP T-junction at a flow rate of 0.25 mL/min. The combined solution entered a FEP capillary submerged in a water bath kept at 80 °C and the product solution was collected externally in a flask. The integrated assembly provided the desired target compounds **6a**–**e** in good overall yields of 73%–77%, among them the local anesthetic AL-12 in its neutral form (entry II). The acidic workup conditions caused near complete conversion into the *Z*-stereoisomer of **6a**–**e** (*Z*:*E* ≥ 9:1) [6].

## 3. Discussion

Even though acetone was the best solvent for the photodecarboxylative addition, the acidic conditions of the subsequent dehydration step led to the formation of its corresponding aldol condensation product, which demanded additional time- and resource-intensive separation. Attempt to use super activated acidic aluminum oxide as a solid catalyst instead failed and showed little to no conversion to the dehydration products **4a**–**e**. The removal of DMF required higher temperatures during evaporation, which caused partial degradation of the photoproducts **3a**–**e** and necessitated subsequent purification. Acetonitrile was thus chosen as the solvent for all three reaction steps as it gave good conversions and could be removed easily. The photodecarboxylations in acetonitrile under flow conditions imposed a somewhat longer residence time of 30 min to reach complete conversions, which was most likely caused by its differing photoactivation mode. Photoreactions of phthalimides in acetonitrile follow a direct excitation pathway, whereas the corresponding transformations in acetone involve triplet-sensitization [23].

The photodecarboxylative addition in the simple in-house flow device required residence times of 20 and 30 min to achieve complete conversions. This respectable performance is best explained by the advanced operation and design features of the microcapillary reactor [24]. The narrow path length, i.e., inner diameter of the capillary, of just 0.8 mm allowed for effective light penetration throughout the reaction mixture. Due to the central position of the 8 W fluorescent tube (inside-out irradiation), all light is furthermore directed onto the reaction capillary, thus resulting in efficient light utilization [25]. However, only approx. ⅓ of the length of the fluorescent tube (23 cm) was covered by the capillary tube (48 windings over 7.8 cm) in the current flow photoreactor, which leaves room for future improvements.

Dehydrations of the photoproducts **3a**–**e** readily furnished the corresponding olefins **4a**–**e** with near complete *E*-selectivity. Using DFT calculations, Kise et al. [26] and independently Li and Janesko [27] showed that the *E*-isomers of *N*-substituted 3-arylmethyleneisoindolin-1-ones are thermodynamically more stable than their *Z*-counterparts. The current results are in line with these findings. Ultrasonic irradiation effectively prevented precipitation of the dehydration products **4a**–**e** and hence clogging, pressure build-up and eventual rupture of the capillary [28].

The one-flow approach required a switch from acidic conditions in the dehydration to basic conditions in the final amination step. Initial attempts to use excess amounts of amine to neutralize the sulfuric acid were unsatisfactory. Instead, a cartridge filled with an ion exchange resin as an acid scavenger was introduced between the thermal loops [29,30]. After several trials, strongly basic Dowex 1-X8 ion exchange resin was found optimal.

Under coupled-flow conditions, amination was achieved rapidly with a residence time of 33 min due to the superior heat transfer inside the small diameter of the capillary [31]. The target *N*-diaminoalkylated 3-arylmethylene-2,3-dihydro-1*H*-isoindolin-1-ones **6a**–**e** were isolated by first converting them into their corresponding hydrochloride salts and subsequent neutralization. This procedure caused near complete isomerization to the *Z*-isomers, presumably via an acylaminium cationic intermediate [32]. The same isomerization was also observed during alternative column chromatographic purification of compounds **6a**–**e** on silica gel [6].

In comparison with the original decoupled three-step synthesis, the developed one-flow protocol gave higher overall yields and furnished the desired compounds **6a**–**e** in shorter time (Table 5). The continuous process only required a single workup, isolation and purification step, which subsequently reduced losses of intermediate products [33]. It should be noted however that the reaction volumes and starting concentrations differed between the two reaction modes. While the overall process time, i.e., the time to pump the complete reaction volume through the entire device, is naturally significantly longer than the actual residence time, flow operation generates the final products **6a**–**e** continuously. The reaction scale can also be increased easily by simply changing the starting reagent volumes.

## 4. Materials and Methods

### 4.1. Experimental Procedures

General experimental details and spectroscopic data can be found in the supporting information. All batch processes and compounds **3a**–**e**, **4a**–**e**, and **6a**–**e** have been previously described in detail. The spectroscopic data are in full agreement with those reported in the literature [6].

#### 4.1.1. Photodecarboxylations under Flow Conditions

The lamp inside the flow photoreactor was allowed to warm up for 10 min. Twenty milliliters of a degassed solution of the respective potassium arylacetate (0.66 mmol) and *N*-(bromoalkyl)phthalimide (0.33 mmol) in acetone-pH 7 buffer or acetonitrile-pH 7 buffer (2:1) were pumped through the flow photoreactor loop. The reaction mixture and 20 mL of fresh organic wash solvent were collected in a flask. After the addition of water (25 mL), most of the organic solvent was removed via rotary evaporation at low temperatures (water bath <30 °C). The remaining reaction mixture was subsequently extracted with CH_2_Cl_2_ (3 × 40 mL) and the combined organic layers were washed with saturated NaHCO_3_ (2 × 40 mL) and brine (1 × 40 mL). After drying over MgSO_4_ and filtration, the reaction mixture was evaporated to dryness on a rotary evaporator at low temperatures (<30 °C) and the solid product obtained was dried in vacuum. When necessary, the crude product was purified by titruration or column chromatography [6].

#### 4.1.2. Coupled Photodecarboxylative Addition and Dehydration under Flow Conditions

The reaction was started as described in Section 4.1.1. After 20 min run time, 20 mL of a 1:1 mixture of acetonitrile and 10 M H_2_SO_4_ were injected into the effluent stream. The combined reaction mixture was pumped through a FEP capillary submerged in an ultrasonic bath. The final product and 30 mL of wash acetonitrile were collected at the exit of the thermal capillary. The collected reaction mixture was diluted with water (25 mL) and extracted with CH_2_Cl_2_ (3 × 25 mL). The combined organic layers were washed with brine (25 mL) and dried over MgSO_4_. Evaporation and drying in vacuum furnished the desired product. When necessary, the crude product was purified by column chromatography [6].

#### 4.1.3. In Series Photodecarboxylative Addition with Thermal Dehydration and Amination under Flow Conditions

The reaction was started as described in Section 4.1.2. The effluent from the thermal ultrasonic reactor loop was passed through a cartridge filled with strongly basic Dowex 1-X8 ion exchange resin (20–50 mesh, ca. 50 mL). After another 20 min run time, a mixture of 2 mL of dialkylamine in 38 mL of acetonitrile was injected into the reaction stream. The combined reaction mixture is passed through a FEP capillary submerged in a water bath at 80 °C. The final product and 60 mL of wash acetonitrile were collected at the exit of the reactor sequence. The collected reaction mixture was diluted with water (50 mL) and acidified using 1 M HCl solution. The solution was subsequently washed with ethyl acetate (50 mL). The aqueous layer containing the target compound (as a hydrochloride salt) was then basified to pH 10 using ammonia solution and extracted with CH_2_Cl_2_ (3 × 25 mL). The combined organic phase was collected, washed with brine (50 mL) and dried over MgSO_4_. The organic layer was evaporated by rotary evaporation and further dried in vacuum. When necessary, the crude product was further purified by column chromatography [6].

## 5. Conclusions

The continuous three-step synthesis of *N*-diaminoalkylated 3-arylmethylene-2,3-dihydro-1*H*-isoindolin-1-ones was successfully realized in a one-flow reactor. Compared to the conventional batch processes, the one-flow system exhibited superior isolated yields. This is mainly caused by a reduction of isolation and purification steps. The developed reaction protocols may be transferred to more advanced and custom-designed continuous flow tandem reactors such as the Vapourtec UV-150 module [8,34]. The process furthermore demonstrates the synthesis potential of arylacetic acids [35] as well as photodecarboxylations [36,37,38].

## Figures and Tables

**Table 1:** Solvent optimization study.

Entry	Solvent	Time [h]	Conversion [%] ^a^
**Photodecarboxylation**			
I	acetone ^b^	3	87
II	acetonitrile ^b^	3	80
III	DMF ^b^	3	45
**Dehydration**			
IV	acetone	5	37 ^c^
V	acetonitrile	5	83
VI	DMF	5	80
**Amination**			
VII	acetone	5	46
VIII	acetonitrile	5	53
IX	DMF	5	55

^a^ Determined by ^1^H-NMR analysis (±3%); ^b^ pH 7 buffer as co-solvent; ^c^ Aldol-condensation product of acetone as a by-product.

**Table 2:** Experimental results for photodecarboxylative addition reactions under flow conditions.

Entry	n	R	Residence Time [min]	Yield of 3 [%]
**pH 7 Buffer-Acetone**				
I	2	H	20	87 (**3a**)
II	2	4-MeO	20	85 (**3b**)
III	3	H	20	95 (**3c**)
IV	3	4-Me	20	80 (**3d**)
V	3	4-F	20	89 (**3e**)
**pH 7 Buffer-Acetonitrile**				
VI	2	H	30	83 (**3a**)
VII	2	4-MeO	30	85 (**3b**)
VIII	3	H	30	91 (**3c**)
IX	3	4-Me	30	83 (**3d**)
X	3	4-F	30	90 (**3e**)

**Table 3:** Experimental results for tandem-photodecarboxylation-dehydration reactions under flow conditions.

Entry	n	R	Residence Times [min] ^a^	Yield of 4 [%] ^b^
I	2	H	30 + 15	91 (**4a**)
II	2	4-MeO	30 + 15	90 (**4b**)
III	3	H	30 + 15	83 (**4c**)
IV	3	4-Me	30 + 15	86 (**4d**)
V	3	4-F	30 + 15	94 (**4e**)

^a^ For photochemical and thermal reaction steps; ^b^ Near complete *E*-selectivity as determined by ^1^H-NMR analysis.

**Table 4:** Experimental results for multistep photodecarboxylation-dehydration-amination reactions under flow conditions.

Entry	n	R	R’	Residence Times [min] ^a^	Yield of 6 [%] ^b^
I	2	H	Et	30 + 15 + 33	75 (**6a**)
II	2	4-MeO	Et	30 + 15 + 33	73 (**6b**)
III	3	H	Me	30 + 15 + 33	75 (**6c**)
IV	3	4-Me	Et	30 + 15 + 33	77 (**6d**)
V	3	4-F	Et	30 + 15 + 33	75 (**6e**)

^a^ For the photochemical and both thermal reaction loops, excluding connecting tubes and cartridge; ^b^ Near complete *Z*-selectivity as determined by ^1^H-NMR analysis.

**Table 5:** Comparison of flow vs. batch performances.

Compound	Batch [7]	Flow
Reaction time^a^	9 h	78 min ^b^
Overall yields of **6a**–**e** [%]	28–45	72–77

^a^ Excluding time required for workup, isolation and purification; ^b^ Overall residence time, excluding connecting tubes and cartridge.

## Supplementary Materials

Technical information on the flow reactor setup, further experimental details and spectroscopic data can be found online.
