# Supplementary material for: Continuous Flow Photochemical and Thermal Multi-Step Synthesis of Bioactive 3-Arylmethylene-2,3-Dihydro-1H-Isoindolin-1-Ones
Source: Molecules. 2019 Dec 11;24(24):4527. doi: 10.3390/molecules24244527 (PMC6943768; doi:10.3390/molecules24244527)
Supplement: Supplementary file 1 [file molecules-24-04527-s001.pdf]

Supplementary Materials for

Continuous Flow Photochemical and Thermal Multi-Step Synthesis of Bioactive 3-Arylmethylene-2,3-Dihydro-1*H*-Isoindolin-1-Ones

Saira Mumtaz [1], Mark J. Robertson [1] and Michael Oelgemöller [1,2,*]

[1] College of Science and Engineering, James Cook University, Townsville, QLD 4811, Australia; saira.mumtaz@my.jcu.edu.au (S.M.); mark.robertson@jcu.edu.au (M.J.R.)

[2] Department of Organic and Macromolecular Chemistry, Ghent University, Krijgslaan 281 S4-bis, 9000 Gent, Belgium

* Correspondence: michael.oelgemoeller@jcu.edu.au; Tel.: +61-7-4781-4543

1. Technical Details

1.1. In-House Photochemical Continuous-Flow Capillary Reactor

**Tube material**	Fluorinated ethylene propylene (FEP), Bohlender GmbH
**External diameter**	1.58 mm
**Internal diameter**	0.8 mm
**Length of tube**	10 m (plus additional ~70 cm on each end)
**Volume of tube**	5 mL (exposed)
**Syringe**	ILS Innovative Labor Systeme GmbH gas-tight syringe (25 mL)
**Pump**	Chemyx syringe pump (Model: Fusion 200)
**Light source**	Ushio G8T5E UVB (300 ± 25 nm; 8 W)

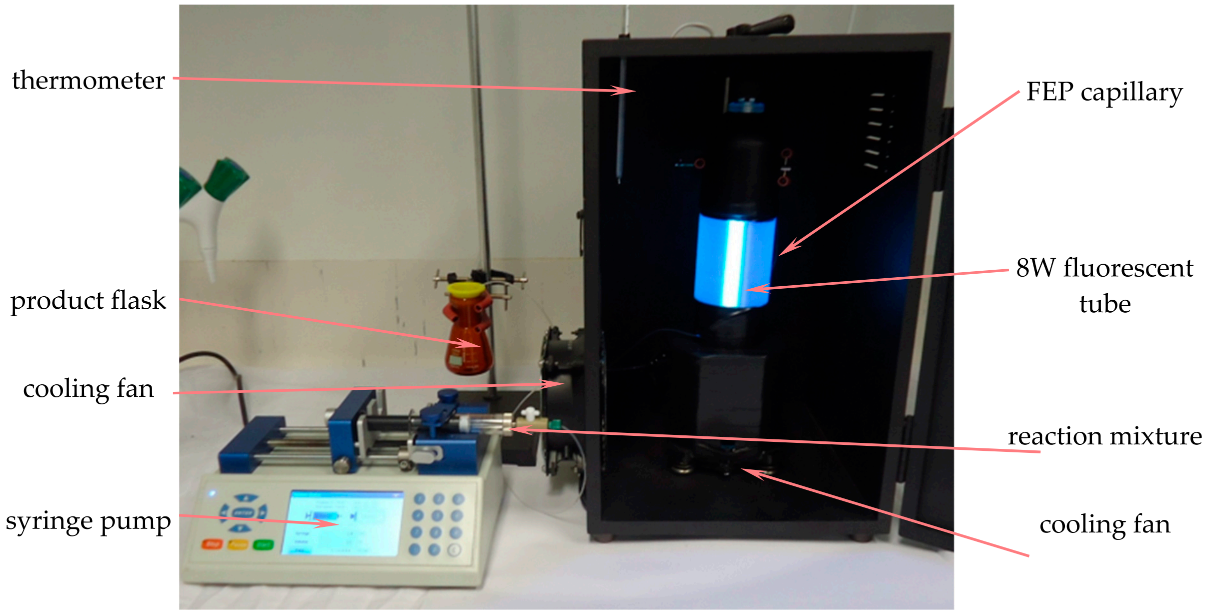

1.2. In-House Triple Photochemical–Thermal–Thermal Continuous-Flow Capillary Reactor

**Tube material**	Fluorinated ethylene propylene (FEP) , Bohlender GmbH
**External diameter**	1.58[a,b]/3.2[c] mm
**Internal diameter**	0.8[a,b]/1.6[b] mm
**Length of tube**	10 m each
**Volume of tube**	5[a,b]/19.6[c] mL
**T-junctions**	Fluorinated ethylene propylene (FEP), Bohlender GmbH
**Syringes**	ILS Innovative Labor Systeme GmbH gas-tight syringes (25 mL)
**Pumps**	Harvard apparatus (Model: 11Plus)[a], kdScientific (Model: KDS-100-CE)[b], Chemyx syringe pump (Model: Fusion 200)[c]
**Light source**	Ushio G8T5E UVB (300 ± 25 nm; 8 W)[a]

[a] Photo loop. [b] Ultrasonic loop. [c] Thermal heating loop.

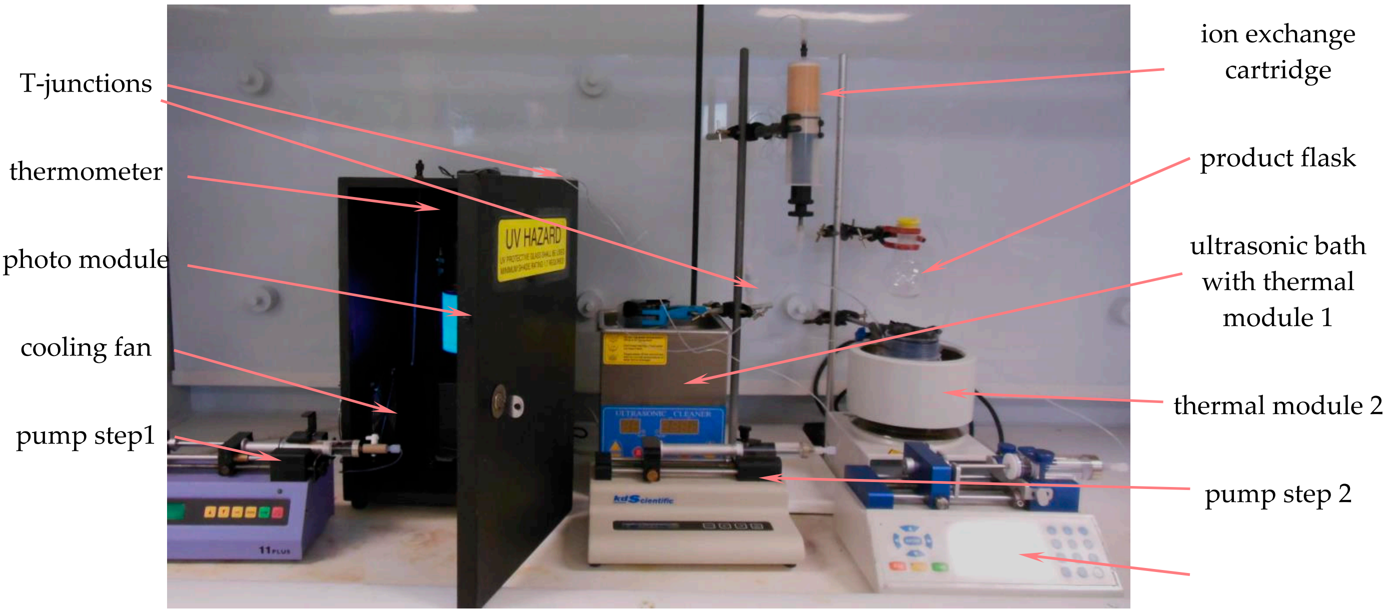

2. Experimental Details

2.1. General Methods

Solvents and Reagents

All solvents and reagents were commercially available (Sigma-Aldrich or Alfa Aesar) and were used without purification.

2.2. Analytical Methods

2.2.1. Melting Point

Melting points were measured using a Tathastu melting point apparatus and are uncorrected.

2.2.2. IR

Infrared spectra were recorded on a Perkin Elmer Spectrum One FT-IR Spectrometer as solids or thin films, and were recorded in the range 600–4000 cm^{-1}. IR peaks are listed in wavenumbers ($\tilde{\nu}$; in cm^{-1}).

2.2.3. NMR

NMR spectra were recorded on an Oxford 300 (^{1}H: 300 MHz and ^{13}C: 75 MHz) with a ^{UNITY}INOVA system console using the Varian Software VnmrJ program or a Bruker AV300 (^{1}H: 300 MHz and ^{13}C: 75 MHz) Nuclear Magnetic Resonance spectrometer with dual probe operating TopSpin 2.1 software. Residual solvent peaks served as an internal standard and samples were prepared in $CDCl_3$ (δ = 7.26/77.3 ppm) or acetone-d_6 (δ = 2.09/30.6 ppm).

2.2.4. FTMS

High resolution mass spectroscopic data were determined on a Bruker BioApex 47 FT mass spectrometer with an electospray (ESI) Analytica of Branford source. Ions were detected in positive mode and/or negative mode within a mass range of *m*/*z* 50–2000. Direct infusion of sample (0.2 mg mL^{-1}) was carried out using a Cole Palmer 74900 syringe pump at a flow rate of 100 μL h^{-1}. N_2 (sourced from a Domnick Hunter UHPLCMS18 Nitrogen Generator, flow of 3 L/min and maintained at 200 °C) was used as the drying gas to assist in desolvation of the droplets produced by ESI from an on axis grounded needle directed to a metal capped nickel coated glass capillary, approximately 1 cm away. All experimental event sequences were controlled and data reduction performed using Bruker Daltonics XMASS ver. 7.0.3.0 software. Detection was in the direct mode from time domain data sets of 512 k (16 scans per experiment). Each spectrum was subjected to zerofill, Gaussian multiplication, and fast Fourier transform and displayed in magnitude mode. The instrument was calibrated using a methanolic solution of sodium trifluoroacetate (0.1 mg/mL MeOH), 200–2000 *m*/*z*.

2.3. Spectroscopic Data

2.3.1. 3-Benzyl-2-(2-Bromoethyl)-3-Hydroxy-2,3-Dihydro-1*H*-Isoindol-1-One (**3a**)

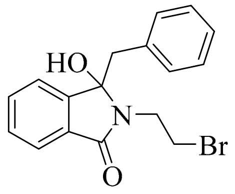

Appearance: colorless crystalline solid.
Melting point: 148 °C.
IR (film): $\tilde{\nu}$ (cm^{-1}) = 3310 br, 3123, 1700, 1611, 1320, and 632.
^{1}H-NMR (300 MHz, $CDCl_3$): δ (ppm): 2.70 (br s, 1 H, OH), 3.14 (d, *J* = 15 Hz, 1H, CH_2Ph), 3.52 (d, *J* = 15 Hz, 1H, CH_2Ph), 3.61–3.82 (m, 3H, CH_2Br, NCH_2), 4.11–4.17 (m, 1H, NCH_2), 6.89 (d, *J* = 9 Hz, 2H, CH), 7.11–7.17 (m, 3H, CH), 7.20–7.23 (d, *J* = 9 Hz, 1H, CH), 7.43–7.58 (m, 2H, CH), 7.62 (d, *J* = 9 Hz, 1H, CH).
^{13}C-NMR (75 MHz, $CDCl_3$): δ (ppm): 29.5 (t, 1C, CH_2Br), 41.4 (t, 1C, CH_2Ph), 43.3 (t, 1C, NCH_2), 91.0 (s, 1C, COH), 122.9 (d, 1C, CH), 123.4 (d, 1C, CH), 127.3 (d, 1C, CH), 128.2 (d, 2C, CH), 129.9 (d, 1C, CH), 130.2 (d, 2C, CH), 130.9 (d, 1C, Cq), 132.3 (d, 1C, CH), 134.3 (s, 1C, Cq), 146.1 (s, 1C, Cq), 167.0 (s, 1C, C=O).
HRMS (ESI/MeOH): *m*/*z*: calcd for $C_{17}H_{16}O_2NBr$ $(M + H)^+$: 346.0437 $(M + Na)^+$ 368.0257, found: 368.0261 ± 1 ppm.

2.3.2. 2-(2-Bromoethyl)-3-Hydroxy-3-(4-Methoxybenzyl)-2,3-Dihydro-1*H*-Isoindol-1-One (**3b**)

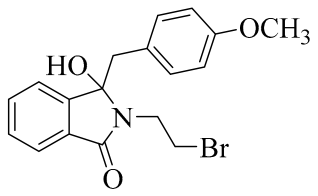

Appearance: pale yellow crystalline solid.
Melting point: 167–168 °C.
IR (film): $\widetilde{\nu}$ (cm^{-1}) = 3379 br, 3106, 1685, 1583, 1366, 1268, and 630.
^{1}H-NMR (300 MHz, CDCl$_3$): δ (ppm): 2.78 (br s, 1H, OH), 3.13 (d, J = 15 Hz, 1H, CH$_2$Ar), 3.49 (d, *J* = 15 Hz, 1H, CH$_2$Ar), 3. 77 (s, 3H, OCH$_3$), 3.72–3.82 (m, 3 H, CH$_2$Br, NCH$_2$), 4.15–4.24 (m, 1H, NCH$_2$), 6.71 (d, *J* = 9 Hz, 2H, CH), 6.84 (d, *J* = 9 Hz, 2H, CH), 7.30 (m, 1H, CH), 7.53 (m, 2H, CH), 7.70 (d, *J* = 9 Hz, 1H, CH).
^{13}C-NMR (75 MHz, acetone-d$_6$): δ (ppm): 29.7 (q, 1C, OCH$_3$), 41.7 (t, 1C, NCH$_2$), 42.5 (t, 1C, CH$_2$Ar), 55.2 (t, 1C, CH$_2$Ph), 91.9 (s, 1C, COH), 113.8 (d, 2C, CH), 123.1 (d, 1C, CH), 123.7 (d, 1C, CH), 127.8 (s, 1C, Cq), 129.8 (d, 1C, CH), 131.8 (d, 2C, CH), 132.5 (d, 1C, Cq), 135.1 (d, 1C, CH), 147.8 (s, 1C, Cq), 159.4 (s, 1C, Cq), 167.0 (s, 1C, C=O).
HRMS (ESI/MeOH): *m*/*z*: calcd for $C_{18}H_{18}NO_3Br$ (M + Na)$^+$: 398.0362, found: 398.0369 ± 2 ppm.

2.3.3. 3-Benzyl-2-(3-Bromopropyl)-3-Hydroxy-2,3-Dihydro-1*H*-Isoindol-1-One (**3c**)

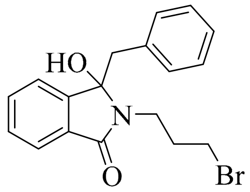

Appearance: colorless crystalline solid.
Melting point: 160 °C.
IR (film): $\widetilde{\nu}$ (cm^{-1}) = 3260 br, 3150, 1680, 1650, 1300, and 685.
^{1}H-NMR (300 MHz, CDCl$_3$): δ (ppm): 2.26–2.45 (m, 2 H, CH$_2$), 2.80 (br s, 1H, OH), 3.13 (d, *J* = 12 Hz, 1H, CH$_2$Ph), 3.48–3.58 (m, 4H, CH$_2$Ph, CH$_2$Br, NCH$_2$), 3.78–3.87 (m, 1H, NCH$_2$), 6.92 (d, *J* = 9 Hz, 2H, CH), 7.19–7.26 (m, 4H, CH), 7.41–7.47 (m, 2H, CH), 7.62 (d, *J* = 9 Hz, 1H, CH).
^{13}C-NMR (75 MHz, CDCl$_3$): δ (ppm): 32.6 (t, 1C, CH$_2$), 33.6 (t, 1C, CH$_2$Br), 39.0 (t, 1C, NCH$_2$), 43.5 (t, 1C, CH$_2$Ph), 90.1 (s, 1C, COH), 123.0 (d, 1C, CH), 123.8 (d, 1C, CH), 127.4 (d, 1C, CH), 128.5 (s, 2C, CH), 130.0 (s, 1C, Cq), 131.0 (d, 2C, CH), 132.3 (d, 1C, CH), 132.7 (d, 1C, CH), 136.2 (s, 1C, Cq), 147.8 (s, 1C, Cq), 167.2 (s, 1C, C=O).
HRMS (ESI/MeOH): *m*/*z*: calcd for $C_{18}H_{18}NO_2Br$ (M + Na)$^+$: 382.0413, found: 382.0435 ± 6 ppm.

2.3.4. 2-(3-Bromopropyl)-3-Hydroxy-3-(4-Methylbenzyl)-2,3-Dihydro-1*H*-Isoindol-1-One (**3d**)

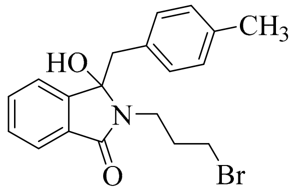

Appearance: colorless crystalline solid.
Melting point: 156–157 °C.
IR (film): $\widetilde{\nu}$ (cm^{-1}) = 3442 br, 3232, 1680, 1600, 1272, and 664.
^{1}H-NMR (300 MHz, CDCl$_3$): δ (ppm): 2.25 (s, 3H, CH$_3$), 2.28–2.43 (m, 3H, CH$_2$, CH$_2$Ar), 3.11 (d, *J* = 12 Hz, 1H, CH$_2$Ar), 3.48–3.57 (m, 4H, CH$_2$Br, NCH$_2$), 6.78 (d, 2H, *J* = 9 Hz, CH), 6.94 (d, 2H, *J* = 9 Hz, CH), 7.18–7.21 (m, 1H, CH), 7.39–7.50 (m, 2H, CH), 7.62 (d, *J* = 9 Hz, 1H, CH), not observed (1H, OH).

^{13}C-NMR (75 MHz, $CDCl_3$): δ (ppm): 20.6 (q, 1C, CH_3), 30.2 (t, 1C, CH_2), 34.7 (t, 1C, CH_2Br), 40.1 (t, 1C, NCH_2), 45.0 (t, 1C, CH_2Ar), 90.3 (s, 1C, COH), 121.3 (d, 2C, CH), 122.7 (d, 1C, CH), 125.0 (d, 2C, CH), 129.0 (s, 1C, Cq), 130.8 (s, 1C, CH), 131.3 (s, 1C, CH), 132.0 (s, 1C, CH), 134.3 (s, 1C, Cq), 135.9 (s, 1C, Cq), 140.4 (s, 1C, Cq), 166.5 (s, 1C, C=O).
HRMS (ESI/MeOH): *m*/*z*: calcd for $C_{19}H_{20}O_2NBr$ $(M + Na)^+$: 396.0570, found: 396.0568 ± 1 ppm.

2.3.5. 2-(3-Bromopropyl)-3-(4-Fluorobenzyl)-3-Hydroxy-2,3-Dihydro-1*H*-Isoindol-1-One (**3e**)

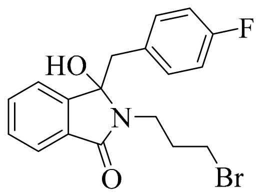

Appearance: yellowish solid.
Melting point: 161 °C.
IR (film): $\widetilde{\nu}$ (cm^{-1}) = 3382 br, 3110, 1690, 1660, 1146, and 680.
^{1}H-NMR (300 MHz, $CDCl_3$): δ (ppm): 2.16–2.44 (m, 2H, CH_2), 3.09 (d, *J* = 12 Hz, 1H, CH_2Ar), 3.47–3.54 (m, 4H, CH_2Ar, CH_2Br, NCH_2), 3.76–3.83 (m, 1H, NCH_2), 6.80–6.89 (m, 4H, CH), 7.11 (d, *J* = 9 Hz, 1H, CH), 7.42–7.47 (m, 2H, CH), 7.61 (d, *J* = 9 Hz, 1H, CH), not observed (1H, OH).
^{13}C-NMR (75 MHz, $CDCl_3$): δ (ppm): 30.0 (t, 1C, CH_2), 34.8 (t, 1C, CH_2Br), 40.2 (t, 1C, NCH_2), 45.5 (t, 1C, CH_2Ar), 93.1 (s, 1C, COH), 120.1 (d, 1C, CH), 122.7 (d, 1C, CH), 124.7 (d, 1C, CH), 128.2 (d, 2C, CH), 130.7 (s, 1C, Cq), 131.3 (d, 2C, CH), 132.9 (d, 1C, CH), 135.7 (s, 1C, Cq), 136.2 (s, 1C, Cq), 158.1 (s, 1C, Cq), 167.0 (s, 1C, C=O).
HRMS (ESI/MeOH): *m*/*z*: calcd for $C_{18}H_{17}O_2NFBr$ $(M + Na)^+$: 400.0319, found: 400.0340 ± 5 ppm.

2.3.6. *E*-3-Benzylidene-2-(2-Bromoethyl)-2,3-Dihydro-1*H*-Isoindol-1-One (**4a**)

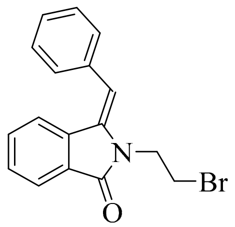

Appearance: yellowish powder.
TLC (SiO_2, ethyl acetate/n-hexane 1:1): R_f = 0.89.
Melting point: 102 °C.
IR (film): $\widetilde{\nu}$ (cm^{-1}) = 3140, 1658, 1620, 1580, 1301, 832, and 689.
^{1}H-NMR (300 MHz, $CDCl_3$): δ (ppm): 3.67 (t, *J* = 6 Hz, 2H, CH_2Br), 4.34 (t, *J* = 6 Hz, 2H, NCH_2), 6.65 (s, 1H, CH), 7.30–7.50 (m, 8H, CH), 7.90 (d, *J* = 9 Hz, 1H, CH).
^{13}C-NMR (75 MHz, $CDCl_3$): δ (ppm): 27.7 (t, 1C, CH_2Br), 41.1 (t, 1C, NCH_2), 110.4 (d, 1C, CH), 123.3 (s, 1C, Cq), 123.4 (d, 2C, CH), 128.9 (d, 2C, CH), 129.3 (d, 1C, CH), 129.6 (d, 1C, CH), 129.6 (d, 1C, CH), 129.9 (d, 1C, CH), 132.0 (s, 1C, Cq), 134.8 (d, 1 C, CH), 135.7 (d, 1C, Cq), 135.8 (s, 1C, Cq), 166.6 (s, 1C, C=O).
HRMS (ESI/MeOH): *m*/*z*: calcd for $C_{17}H_{14}ONBr$ $(M + Na)^+$: 350.0151, found: 350.0149 ± 1 ppm.

2.3.7. *E*-2-(2-Bromoethyl)-3-(4-Methoxybenzylidene)-2,3-Dihydro-1*H*-Isoindol-1-One (**4b**)

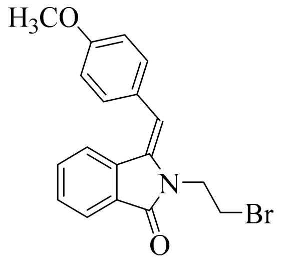

Appearance: yellow oil.
TLC (SiO_2, ethyl acetate/n-hexane 1:1): R_f = 0.74.
IR (film): $\widetilde{\nu}$ (cm^{-1}) = 3126, 1685, 1583, 1581, 1366, 1268, 826, and 630.
^{1}H-NMR (300 MHz, $CDCl_3$): δ (ppm): 3.66 (t, *J* = 9 Hz, 2H, CH_2Br), 3.93 (s, 3H, OCH_3), 4.33 (t, *J* = 9 Hz, 2H, NCH_2), 6.60 (s, 1H, CH), 7.02 (d, *J* = 9 Hz, 2H, CH), 7.40–7.43 (m, 5H, CH), 7.90 (d, *J* = 9 Hz, 1H, CH).
^{13}C-NMR (75 MHz, $CDCl_3$): δ (ppm): 27.8 (t, 1C, CH_2Br), 42.1 (t, 1C, NCH_2), 55.2 (q, 1C, OCH_3), 107.0 (d, 1C, CH), 114.3 (d, 2C, CH), 123.5 (d, 1C, CH), 123.7 (s, 1C, Cq), 127.0 (s, 1C, Cq), 128.2 (d, 1C, CH), 129.4 (d, 1C, CH), 130.9 (d, 2C, CH), 131.9 (s, 1C, Cq), 134.4 (d, 1C, CH), 134.3 (s, 1C, Cq), 159.6 (s, 1C, Cq), 166.6 (s, 1C, C=O).
HRMS (ESI/MeOH): *m*/*z*: calcd for $C_{18}H_{16}O_2NBr$ $(M + Na)^+$: 380.0257, found: 380.0252 ± 1 ppm.

2.3.8. *E*-3-Benzylidene-2-(3-Bromopropyl)-2,3-Dihydro-1*H*-Isoindol-1-One (**4c**)

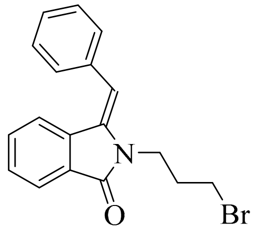

Appearance: pale yellow solid.
TLC (SiO_2, ethyl acetate/n-hexane 1:1): R_f = 0.91.
Melting point: 127 °C.
IR (film): $\widetilde{\nu}$ (cm^{-1}) = 3148, 1633, 1663, 1574, 1327, 841, and 680.
^{1}H-NMR (300 MHz, $CDCl_3$): δ (ppm): 2.34 (m, 2H, CH_2), 3.52 (t, *J* = 9 Hz, 2H, CH_2Br), 4.05 (t, *J* = 9 Hz, 2H, NCH_2), 6.60 (s, 1H, CH), 7.29–7.32 (m, 2H, CH), 7.42–7.46 (m, 6H, CH), 7.83 (d, J = 9 Hz, 1H, CH).
^{13}C-NMR (75 MHz, $CDCl_3$): δ (ppm): 30.7 (t, 1C, CH_2), 31.5 (t, 1C, CH_2Br), 38.1 (t, 1C, NCH_2), 110.5 (d, 1C, CH), 123.2 (d, 1 C, CH), 123.3 (s, 1C, Cq), 128.0 (d, 1C, CH), 128.3 (s, 1C, Cq), 128.4 (d, 2C, CH), 128.8 (d, 1C, CH), 129.6 (d, 2C, Cq), 130.1 (s, 1C, Cq), 131.7 (d, 1C, CH), 135.0 (s, 1C, Cq), 136.1 (s, 1C, Cq), 166.8 (s, 1C, C=O).
HRMS (ESI/MeOH): *m*/*z*: calcd for $C_{18}H_{16}NOBr$ $(M + Na)^+$: 364.0307, found: 364.0316 ± 3 ppm.

2.3.9. *E*-2-(3-Bromopropyl)-3-(4-Methylbenzylidene)-2,3-Dihydro-1*H*-Isoindol-1-One (**4d**)

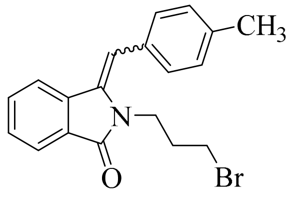

Appearance: yellow oil.
IR (film): $\widetilde{\nu}$ (cm^{-1}) = 3101, 1679, 1602, 1581, 1372, 846, and 669.
^{1}H-NMR (300 MHz, $CDCl_3$): δ (ppm): 2.31–2.36 (m, 2H, CH_2), 2.43 (s, 3H, CH_3), 3.51 (t, *J* = 6 Hz, 2H, CH_2Br), 4.04 (t, *J* = 6 Hz, 2H, NCH_2), 6.58 (s, 1H, CH), 7.23–7.29 (m, 1H, CH), 7.32–7.45 (m, 6H, CH), 7.83 (d, *J* = 6 Hz, 1H, CH).

^{13}C-NMR (75 MHz, $CDCl_3$): δ (ppm): 21.3 (q, 1C, CH_3), 28.3 (t, 1C, CH_2), 30.6 (t, 1C, CH_2Br), 41.3 (t, 1C, NCH_2), 107.1 (d, 1C, CH), 114.2 (d, 1C, CH), 119.1 (d, 1C, CH), 123.0 (d, 1C, CH), 123.5 (d, 1C, Cq), 127.9 (d, 1C, CH), 129.2 (d, 1C, CH), 130.9 (d, 1C, CH), 131.3 (d, 1C, CH), 131.6 (d, 1C, Cq), 134.5 (d, 1C, CH), 135.7 (s, 1C, Cq), 138.0 (s, 1C, Cq), 141.4 (s, 1C, Cq), 166.5 (s, 1C, C=O).
HRMS (ESI/MeOH): *m*/*z*: calcd for $C_{19}H_{18}NOBr$ $(M + Na)^+$: 378.0464, found: 378.0471 ± 2 ppm.

2.3.10. *E*-2-(3-Bromopropyl)-3-(4-Fluorobenzylidene)-2,3-Dihydro-1*H*-Isoindol-1-One (**4e**)

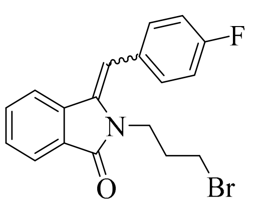

Appearance: yellow oil.
IR (film): $\tilde{\nu}$ (cm^{-1}) = 3129, 1654, 1663, 1582, 1151, 825, and 683.
^{1}H-NMR (300 MHz, $CDCl_3$): δ (ppm): 2.33 (m, 2 H, CH_2), 3.51 (t, *J* = 6 Hz, 2 H, CH_2Br), 4.04 (t, *J* = 6 Hz, 2 H, NCH_2), 6.54 (s, 1 H, CH), 7.14 (dd, *J* = 6, 3 Hz, 2 H, CH), 7.31–7.47 (m, 5 H, CH), 7.84 (d, *J* = 6 Hz, 1 H, CH).
^{13}C-NMR (75 MHz, $CDCl_3$): δ (ppm): 27.8 (t, 1 C, CH_2), 30.6 (t, 1 C, CH_2Br), 39.3 (t, 1 C, NCH_2), 110.9 (d, 1 C, CH), 114.6 (d, 1 C, CH), 119.4 (d, 1 C, CH), 123.1 (d, 1 C, CH), 126.9 (d, 1 C, Cq), 129.1 (d, 1 C, CH), 129.4 (d, 1 C, CH), 129.8 (d, 1 C, CH), 130.9 (d, 1 C, CH), 131.7 (d, 1 C, Cq), 132.3 (d, 1 C, CH), 135.1 (s, 1 C, Cq), 138.4 (s, 1 C, Cq), 159.5 (s, 1 C, Cq), 167.8 (s, 1 C, C=O).
HRMS (ESI/MeOH): *m*/*z*: calcd for $C_{18}H_{15}NOBrF$ $(M + Na)^+$: 382.0213, found: 382.0215 ± 1 ppm.

2.3.11. *Z*-3-Benzylidene-2-[2-(Diethylamino)Ethyl]-2,3-Dihydro-1*H*-Isoindol-1-One (**6a**)

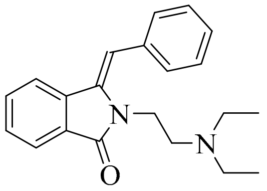

Appearance: beige solid.
Melting point: 43 °C.
IR (film): $\tilde{\nu}$ (cm^{-1}) = 3132, 1645, 1612, 1574, 1300, 824, and 691.
^{1}H-NMR (300 MHz, $CDCl_3$): δ (ppm): 1.13 (t, *J* = 9 Hz, 6H, 2 × CH_3), 2.67–2.74 (m, 4H, $N(CH_2)_2$), 2.83 (t, *J* = 9 Hz, 2H, NCH_2), 4.04 (t, *J* = 9 Hz, 2H, NCH_2), 6.68 (s, 1H, CH), 7.33–7.49 (m, 8H, CH), 7.87 (d, *J* = 9 Hz, 1H, CH).
^{13}C-NMR (mixture of stereoisomers, 75 MHz, $CDCl_3$): δ (ppm): 12.1 (q, 2C, CH_3), 39.2 (t, 1C, CH_2N), 47.1 (t, 2C, $N(CH_2)_2$), 50.3 (t, 1C, NCH_2), 106.4 (d, 1C, CH), 119.3 (d, 1C, CH), 123.3 (s, 1C, Cq), 127.7 (d, 1C, CH), 128.4 (d, 2C, CH), 128.5 (d, 1C, CH), 129.0 (d, 2C, CH), 129.8 (d, 1C, CH), 131.9 (d, 1C, CH), 134.9 (s, 1C, Cq), 135.0 (s, 1C, Cq), 138.6 (s, 1C, Cq), 169.0 (s, 1C, C=O).
HRMS (ESI/MeOH): *m*/*z*: calcd for $C_{21}H_{24}ON_2$ $(M + Na)^+$: 343.1781, found: 343.1792 ± 3 ppm.

2.3.12. *Z*-2-[2-(Diethylamino)Ethyl]-3-(4-Methoxybenzylidene)-2,3-Dihydro-1*H*-Isoindol-1-One (**6b**)

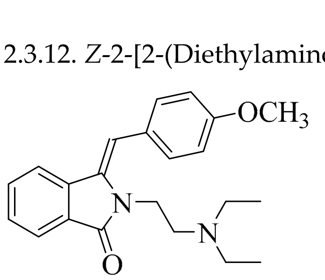

Appearance: beige oil.
IR (film): $\tilde{\nu}$ (cm^{-1}) = 3130, 1679, 1589, 1371, 1268, 830, and 680.

^{1}H-NMR (300 MHz, CDCl$_3$): δ (ppm): 1.12 (t, *J* = 9 Hz, 6H, 2 × CH$_3$), 2.65–2.72 (m, 4H, N(CH$_2$)$_2$), 2.81 (t, *J* = 9 Hz, 2H, NCH$_2$), 3.92 (s, 3H, OCH$_3$), 4.02 (t, *J* = 9 Hz, 2H, NCH$_2$), 6.61 (s, 1H, CH), 7.01 (d, *J* = 9 Hz, 2H, CH), 7.40–7.45 (m, 5H, CH), 7.87 (d, *J* = 9 Hz, 1H, CH).
^{13}C-NMR (75 MHz, CDCl$_3$): δ (ppm): 12.0 (q, 2C, CH$_3$), 38.0 (t, 1C, CH$_2$N), 47.2 (t, 2C, N(CH$_2$)$_2$), 50.3 (t, 1C, NCH$_2$), 55.4 (q, 1C, OCH$_3$), 106.4 (d, 1C, CH), 119.1 (d, 1C, CH), 123.1 (s, 1C, Cq), 127.0 (d, 1C, CH), 128.8 (d, 2C, CH), 129.1 (d, 1C, CH), 130.4 (d, 2C, CH), 131.9 (s, 1C, Cq), 134.4 (d, 1C, CH), 135.8 (s, 1C, Cq), 138.7 (s, 1C, Cq), 159.3 (s, 1C, Cq), 166.5 (s, 1C, C=O).
HRMS (ESI/MeOH): *m*/*z*: calcd for $C_{22}H_{26}O_2N_2$ (M + H)$^+$: 351.2067, found: 351.2061 ± 2 ppm. calcd for $C_{22}H_{26}O_2N_2$ (M + Na)$^+$: 373.1886, found: 373.1886 ± 0 ppm.

2.3.13. *Z*-3-Benzylidene-2-[3-(Dimethylamino)Propyl]-2,3-Dihydro-1*H*-Isoindol-1-One (**6c**)

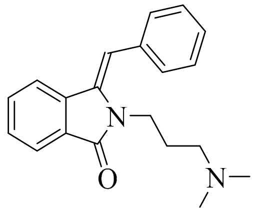

Appearance: beige oil.
IR (film): $\widetilde{\nu}$ (cm^{-1}) = 3138, 1640, 1649, 1581, 1329, 852, and 678.
^{1}H-NMR (300 MHz, acetone-d$_6$): δ (ppm): 1.91–2.01 (m, 2H, CH$_2$), 2.28 (s, 6H, N(CH$_3$)$_2$), 2.43 (t, *J* = 9 Hz, 2H, NCH$_2$), 3.98 (t, *J* = 9 Hz, 2H, NCH$_2$), 6.67 (s, 1H, CH), 7.30–7.48 (m, 8H, CH), 7.87 (d, *J* = 9 Hz, 1H, CH).
^{13}C-NMR (75 MHz, CDCl$_3$): δ (ppm): 26.7 (t, 1C, CH$_2$), 37.6 (t, 1C, NCH$_2$), 45.5 (t, 2C, N(CH$_3$)$_2$), 56.9 (t, 1C, CH$_2$N), 110.4 (d, 1C, CH), 123.1 (d, 1C, CH), 123.2 (s, 1C, Cq), 127.8 (d, 1C, CH), 128.7 (d, 1C, CH), 129.2 (d, 2C, CH), 129.6 (d, 1C, CH), 129.9 (s, 1C, Cq), 130.4 (d, 1C, CH), 131.4 (d, 1C, CH), 135.1 (d, 1C, CH), 135.4 (s, 1C, Cq), 136.4 (s, 1C, Cq), 166.7 (s, 1C, C=O).
HRMS (ESI/MeOH): *m*/*z*: calcd for $C_{20}H_{22}ON_2$ (M + Na)$^+$: 329.1624, found: 329.1624 ± 1 ppm.

2.3.14. *Z*-2-[2-(Diethylamino)Propyl]-3-(4-Fluorobenzylidene)-2,3-Dihydro-1*H*-Isoindol-1-One (**6d**)

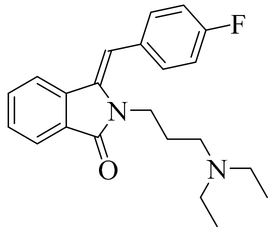

Appearance: beige solid.
Melting point: 40 °C.
IR (film): $\widetilde{\nu}$ (cm^{-1}) = 3230, 1671, 1648, 1576, 1148, 836, and 671.
^{1}H-NMR (300 MHz, acetone-d$_6$): δ (ppm): 1.02 (t, *J* = 6 Hz, 6H, 2 × CH$_3$), 1.90 (t, *J* = 6 Hz, 2H, CH$_2$), 2.54 (t, *J* = 6 Hz, 6H, N(CH$_2$)$_2$, NCH$_2$), 3.92 (t, *J* = 9 Hz, 2H, NCH$_2$), 6.54 (s, 1H, CH), 7.12 (t, *J* = 9 Hz, 2 H, CH), 7.21 (d, *J* = 9 Hz, 1H, CH), 7.30–7.44 (m, 4H, CH), 7.83 (d, *J* = 9 Hz, 1H, CH).
^{13}C-NMR (75 MHz, CDCl$_3$): δ (ppm): 11.6 (q, 2C, CH$_3$), 25.7 (t, 1C, CH$_2$), 38.0 (t, 1C, CH$_2$N), 46.3 (t, 2C, N(CH$_2$)$_2$), 50.5 (t, 1C, NCH$_2$), 106.9 (d, 1C, CH), 110.7 (d, 1C, CH), 114.5 (s, 1C, Cq), 119.7 (d, 1C, CH), 127.5 (d, 1C, CH), 128.3 (d, 2C, CH), 130.4 (d, 1C, CH), 131.2 (s, 1C, Cq), 131.7 (s, 1C, Cq), 134.0 (d, 1C, CH), 135.8 (d, 1C, CH), 138.9 (s, 1C, Cq), 158.5 (s, 1C, Cq), 167.1 (s, 1C, C=O).
HRMS (ESI/MeOH): *m*/*z*: calcd for $C_{22}H_{25}N_2OF$ (M + H)$^+$: 353.2024, found: 353.2031 ± 2 ppm.

2.3.15. *Z*-2-[2-(Diethylamino)Propyl]-3-(4-Methylbenzylidene)-2,3-Dihydro-1*H*-Isoindol-1-One (**6e**)

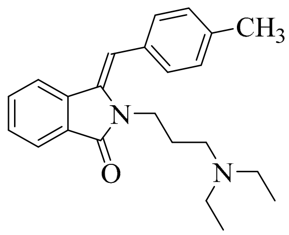

Appearance: beige oil.
IR (film): $\widetilde{\nu}$ (cm^{-1}) = 3101, 1682, 1612, 1565, 1361, 850, and 673.
^{1}H-NMR (300 MHz, CDCl$_3$): δ (ppm): 1.02 (t, *J* = 6 Hz, 6H, 2 × CH$_3$), 1.91 (m, 2H, CH$_2$), 2.42 (s, 3H, CH$_3$), 2.52–2.59 (m, 6H, N(CH$_2$)$_2$, NCH$_2$), 3.93 (t, *J* = 9 Hz, 2H, NCH$_2$), 6.58 (s, 1H, CH), 7.23 (d, *J* = 9 Hz, 2H, CH), 7.29–7.36 (m, 4H, CH), 7.40–7.43 (m, 1H, CH), 7.82 (d, *J* = 9 Hz, 1H, CH).
^{13}C-NMR (75 MHz, CDCl$_3$): δ (ppm): 11.9 (q, 2C, CH$_3$), 19.5 (q, 1C, CH$_3$), 25.7 (t, 1C, CH$_2$), 38.8 (t, 1C, CH$_2$N), 43.7 (t, 2C, N(CH$_2$)$_2$), 51.3 (t, 1C, NCH$_2$), 106.1 (d, 1C, CH), 111.2 (d, 1C, CH), 114.0 (s, 1C, Cq), 120.3 (d, 1C, CH), 127.0 (d, 1C, CH), 128.4 (d, 2C, CH), 130.9 (d, 1C, CH), 131.1 (s, 1C, Cq), 131.7 (s, 1C, Cq), 134.4 (d, 1C, CH), 135.0 (d, 1C, CH), 138.7 (s, 1C, Cq), 140.1 (s, 1C, Cq), 166.7 (s, 1C, C=O).
HRMS (ESI/MeOH): *m*/*z*: calcd for $C_{23}H_{28}N_2O$ (M + Na)$^+$: 371.2094, found: 371.2103 ± 3 ppm.
